# Giant thyroglossal duct cyst in an adult: CT findings and differential diagnosis

**DOI:** 10.1016/j.radcr.2026.02.047

**Published:** 2026-03-25

**Authors:** Drissia Benfadil, Mohamed Bouallou, Achraf Amine Sbai, Azeddine Lachkar, Fahd El Ayoubi El Idrissi

**Affiliations:** a Faculty of Medicine and Pharmacy, Mohammed first University, Oujda, Morocco; b Department of Otorhinolaryngology, Mohammed VI University Hospital, Mohammed first University, Oujda, Morocco; c Laboratory of Oto-Neuro-Ophthalmology (LORNO), Faculty of Medicine and Pharmacy, Mohammed first University, Oujda, Morocco; d Mohammed First University, LAMCESM, Oujda, Morocco

**Keywords:** Thyroglossal duct cyst, Adult, Sistrunk procedure, Neck mass, Computed tomography

## Abstract

Thyroglossal duct cysts are the most common congenital cervical anomalies arising from incomplete obliteration of the thyroglossal tract. They are typically diagnosed in childhood and rarely present in adults. Giant TGDCs in adults are exceptionally rare and can mimic thyroid or other midline neck pathologies, posing diagnostic challenge. We report the case of a 37-year-old woman with a 5-year history of a progressively enlarging, elastic, and mobile midline neck mass associated with mild dysphagia. Physical examination revealed a soft, infra-hyoid, midline swelling measuring approximately 6 cm. Thyroid function tests were normal. Contrast-enhanced computed tomography demonstrated a well-circumscribed, homogeneous, non-enhancing cystic lesion consistent with a thyroglossal duct cyst. The patient underwent surgical excision via the Sistrunk procedure. Intraoperatively, a thin-walled cyst adherent to the central hyoid segment was excised en bloc. Histopathological analysis confirmed a benign thyroglossal duct cyst. The postoperative course was uneventful, and no recurrence was observed during 1-year follow-up. This case highlights the diagnostic value of cross-sectional imaging in the evaluation of atypically large midline neck masses and its role in guiding safe and effective surgical planning.

## Introduction

TGDCs represent the most common congenital cervical mass of embryologic origin, typically located in the anterior midline of the neck. Both males and females are equally affected [1]. This anomaly occurs in approximately 7% of people [2]. Most TGDCs exhibit slow, progressive growth, typically measuring between 2 and 4 cm in diameter.

Although TGDCs are most frequently diagnosed in childhood, a substantial proportion of cases may remain undetected until adulthood, where the differential diagnosis of midline neck masses is broader and often requires imaging for accurate characterization [3]. They typically manifest as painless midline neck masses that exhibit mobility during swallowing and tongue protrusion, most commonly situated inferior to the hyoid bone [4].

Cross-sectional imaging, particularly computed tomography, plays an important role in adults by confirming the cystic nature of the lesion, defining its anatomical relationships, and excluding alternative diagnoses or complications. The treatment of choice is surgical excision via the Sistrunk procedure [5], which entails the complete removal of the epithelial tract extending from the foramen cecum to the cyst.

We report a case of an unusually large TGDC in an adult, emphasizing the imaging features and diagnostic considerations relevant to clinical practice.

## Case presentation

A 37-year-old female presented to our department with a 5-year history of a progressively enlarging, elastic, and freely mobile midline neck swelling associated with mild dysphagia. There was no history of radiation exposure, no personal or family history of thyroid disease or malignancy, and no symptoms suggestive of hypo or hyperthyroidism.

**Fig. 1:**
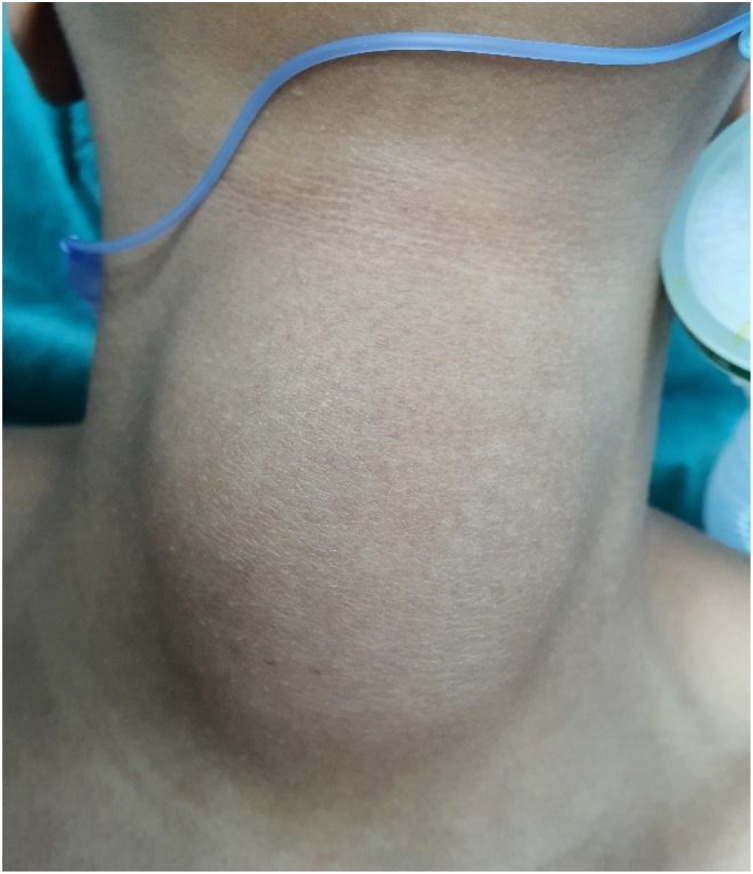
Preoperative photograph showing a large midline neck mass.

Physical examination revealed a large, anterior, infra-hyoid midline mass, soft and fluctuant on palpation, measuring approximately 6 cm in its greatest dimension (Fig. 1). The overlying skin was normal, with no erythema, warmth, or fistulous opening. The lesion was non-tender and moved with tongue protrusion. Thyroid palpation was limited due to the size and location of the mass, which partially obscured the gland. No cervical lymphadenopathy was detected. Flexible nasofibroscopy revealed no abnormality of the upper aerodigestive tract. The remainder of the head and neck examination was unremarkable.

Routine laboratory investigations, including complete blood count, inflammatory markers, and serum electrolytes, were within normal limits. Thyroid function tests confirmed a euthyroid state.

**Fig. 2:**
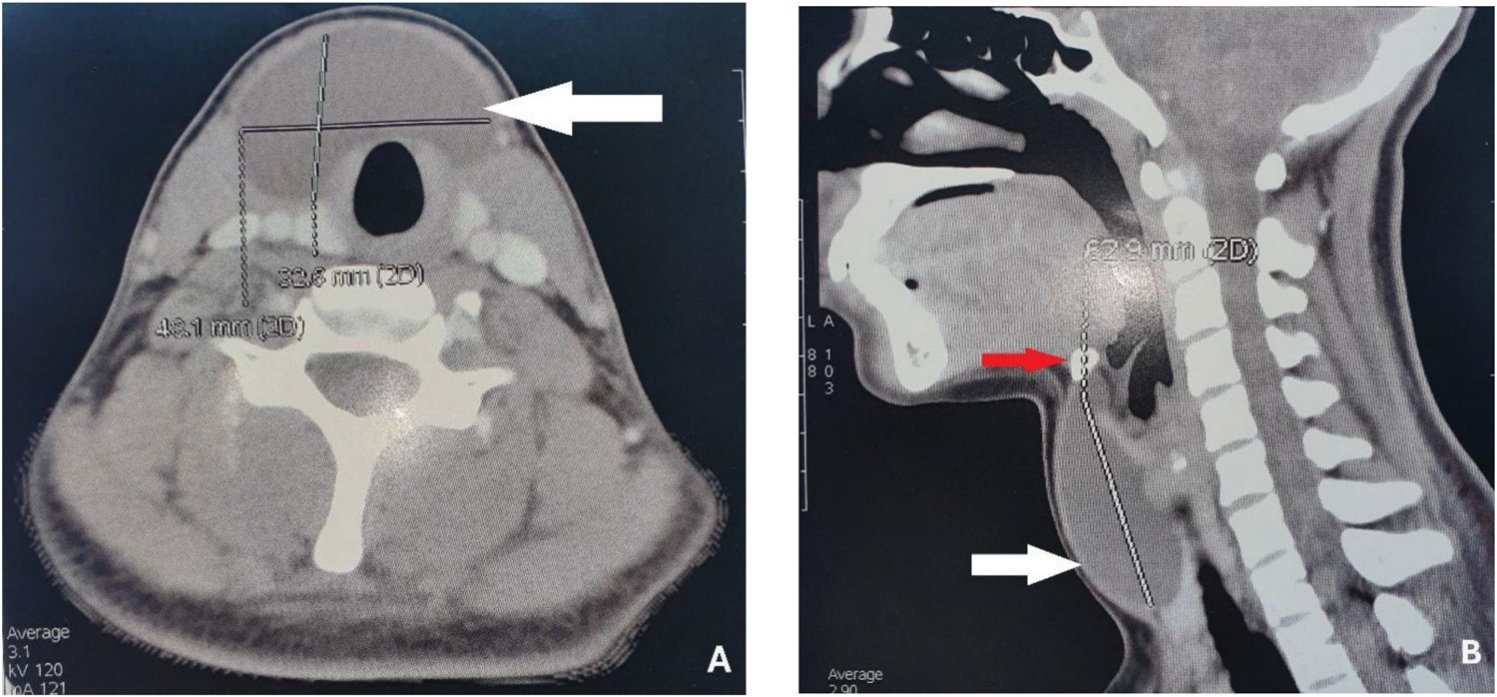
Contrast-enhanced computed tomography of the neck, parenchymal window, axial (A) and sagittal (B) reconstructions, showing a well-defined, homogeneous midline cystic lesion (white arrow) measuring approximately 6.3 cm in maximal diameter, located inferior to the hyoid bone (red arrow). No internal septations, solid components, or post-contrast enhancement are identified.

Contrast-enhanced CT of the neck demonstrated a well-circumscribed, homogeneous, non-enhancing cystic lesion located along the midline in the infrahyoid region, measuring approximately 6.3 × 4.8 × 3.26 cm (Fig. 2). The thyroid gland was normally located and appeared unremarkable. There was no evidence of invasion, calcification, or solid components. Based on clinical and radiological features, a diagnosis of TGDC was established.

**Fig. 3:**
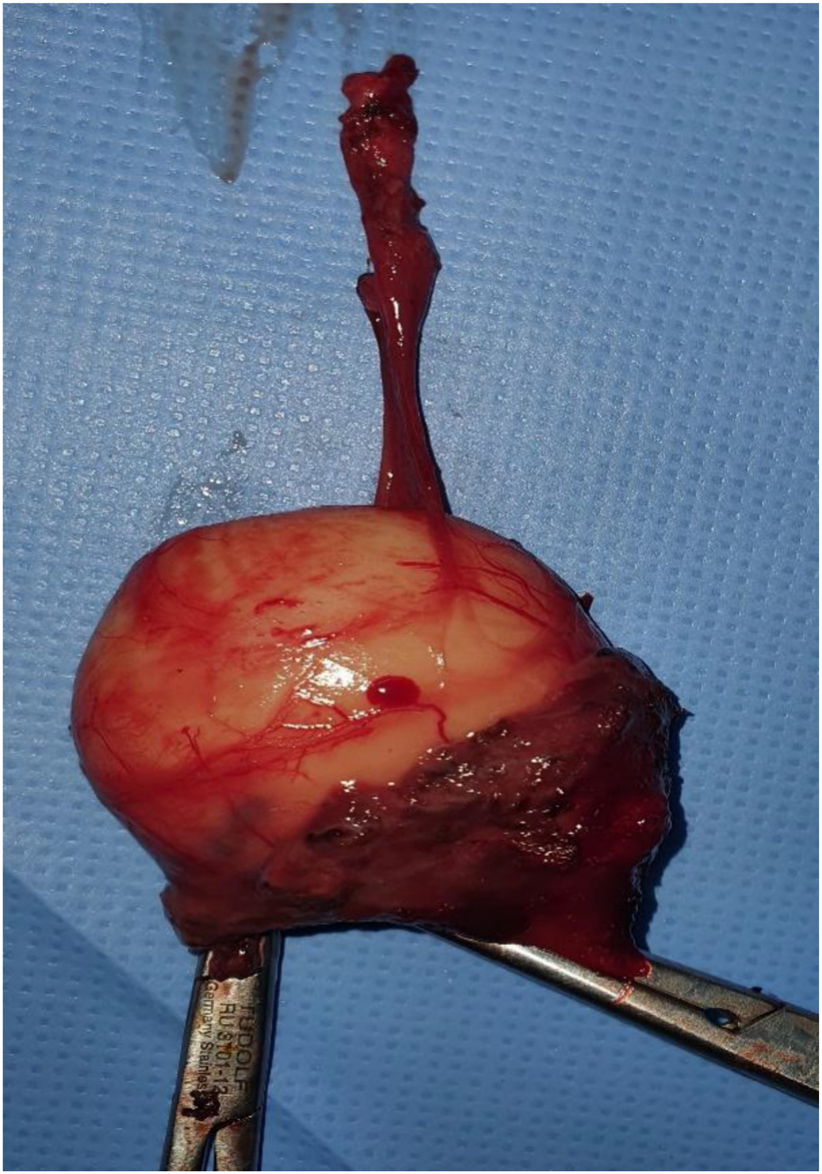
Photograph of the excised thyroglossal duct cyst specimen.

The patient underwent a Sistrunk procedure under general anesthesia, which involved en bloc excision of the cyst, the central portion of the hyoid bone, and the tract up to the foramen cecum. Intraoperatively, a well-defined, thin-walled cystic mass was identified, strongly adherent to the central hyoid segment but easily separable from surrounding structures. The specimen weighed 117.5 grams and measured approximately 6 cm in diameter (Fig. 3). Histopathological examination confirmed the TGDC without any evidence of dysplasia or malignancy.

The postoperative course was uneventful, with no complications observed. The patient was discharged on postoperative day 3. Follow-up at 1 year revealed no evidence of recurrence or functional complications.

## Discussion

**Fig. 4:**
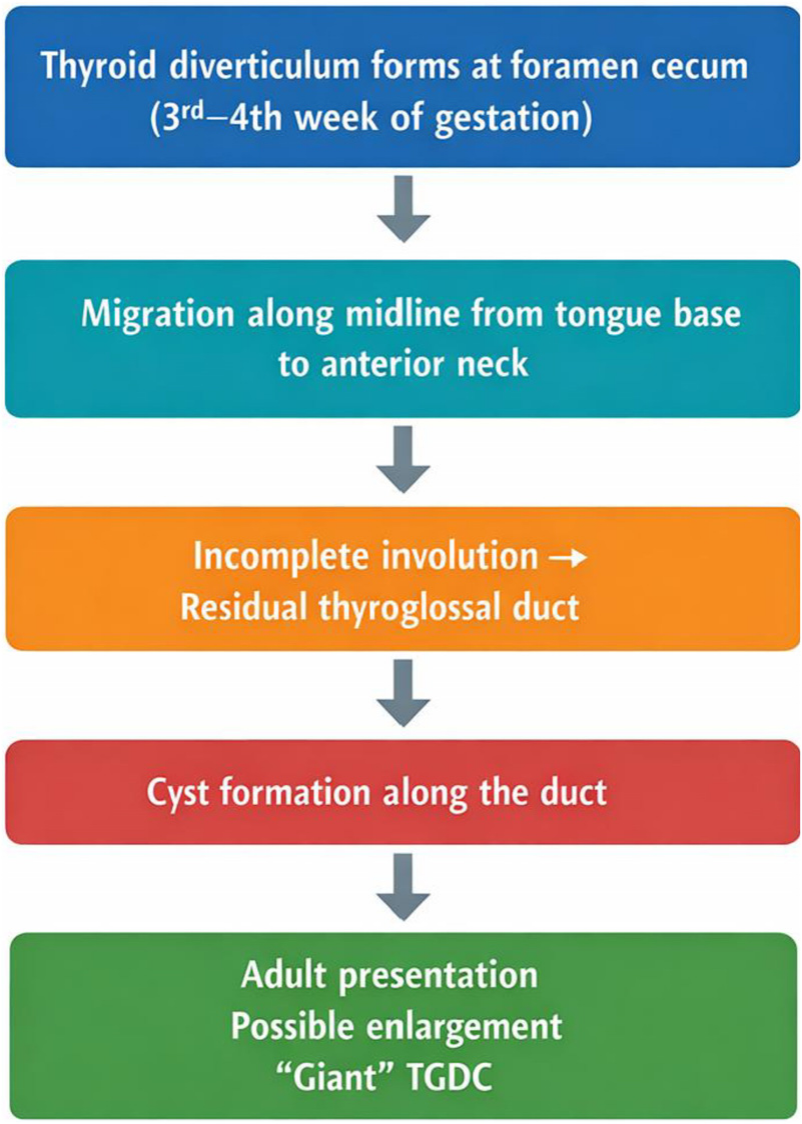
Pathogenesis of thyroglossal duct cysts.

The thyroid gland originates from an endodermal swelling in the pharyngeal floor between the first and second pharyngeal pouches, around the 24th day of embryogenesis [6]. During its descent anterior to the hyoid bone, the thyroid primordium remains connected to the tongue via the thyroglossal duct, which normally involutes by the tenth week of gestation. Failure of this involution may result in the persistence of epithelial remnants, predisposing to thyroglossal duct cyst formation. This embryologic mechanism explains the characteristic midline distribution of TGDCs and their potential occurrence anywhere along the tract of thyroid descent (Fig. 4).

Although there is no universally accepted definition of a ‘giant’ TGDC, several authors in the literature have used this term for lesions exceeding 5 cm in greatest dimension in adult patients [4,6]. Based on this commonly used criterion, the present lesion, measuring 6.3 cm in maximum diameter, fulfills the definition of a giant TGDC, thereby supporting the novelty of this case.

TGDCs are the most frequent congenital midline cervical anomalies in the pediatric population, particularly during the first decade of life, accounting for up to 70% of all congenital neck masses, with an estimated prevalence of approximately 7% in the general population [2,7]. In contrast, adult presentation is uncommon and often associated with diagnostic uncertainty due to a broader differential diagnosis of anterior neck masses.

Although TGDCs typically present in the first 2 decades of life, their presentation in adulthood is exceptional and frequently delayed, as observed in our 37-year-old patient.

TGDCs can develop anywhere along the embryologic descent of the thyroid gland; from the foramen cecum at the tongue base to the suprasternal notch; accounting for their variable clinical presentation. They classically appear as a painless, fluctuant, midline swelling that moves upward with deglutition and tongue protrusion. It is often associated with dysphagia [8]. However, in large or atypical lesions, these characteristic clinical signs may be obscured, increasing the risk of misdiagnosis. Compressive symptoms such as dysphagia, dyspnea, or voice changes may occur due to mass effect on adjacent aerodigestive structures.

In our case, the lesion was located in the infrahyoid region and caused mild dysphagia. Its atypical size and position posed a diagnostic challenge, as it clinically resembled a thyroid mass. The absence of airway compromise or thyroid dysfunction, further contributed to diagnostic uncertainty. This initially raised the suspicion of a TGDC, underscoring the pivotal role of imaging in establishing the correct diagnosis.

Imaging plays a pivotal role in characterizing TGDCs and differentiating them from other midline neck lesions. CT of the neck remains the cornerstone imaging modality for evaluating cervical masses in adults, offering superior anatomical resolution and accurate delineation of lesion boundaries and adjacent structures [3]. In our case, cross-sectional imaging demonstrated a well-circumscribed, homogeneous midline cystic lesion without contrast enhancement, findings highly suggestive of a thyroglossal duct cyst.

However, in selected cases, advanced magnetic resonance imaging sequences; particularly diffusion-weighted and dynamic contrast-enhanced studies; can provide complementary diagnostic information, especially when vascular malformations, abscesses, or metastatic lymphadenopathy are suspected [9].

Typically, TGDCs appear as well-defined, non-enhancing, homogeneous cystic lesions located along the midline, most commonly in the infrahyoid region, as observed in our patient [10]. Conversely, thyroid ultrasonography is more commonly utilized in pediatric populations owing to its non-ionizing nature, ease of access, and enhanced characterization of thyroid parenchyma.

In addition to TGDCs, several congenital cystic lesions should be considered in the imaging differential diagnosis of anterior neck masses in adults. Branchial cleft cysts represent a common congenital cystic anomaly and typically arise laterally, along the anterior border of the sternocleidomastoid muscle [11]. On cross-sectional imaging, they usually appear as well-circumscribed, non-enhancing cystic lesions located lateral to the carotid space. However, when large, branchial cleft cysts may extend medially or inferiorly, potentially simulating a midline lesion and complicating differentiation from a TGDC. In the present case, the strictly midline infrahyoid location and intimate relationship with the hyoid bone favored a TGDC rather than a branchial cleft origin.

Dermoid and epidermoid cysts constitute another important group of congenital midline cystic lesions. Dermoid cysts typically contain fat or lipid-rich material and may demonstrate negative attenuation values on CT, often with a heterogeneous internal appearance [12]. Epidermoid cysts, in contrast, usually show homogeneous fluid attenuation but frequently exhibit diffusion restriction on MRI. These imaging characteristics, along with their usual submental or suprahyoid location, help distinguish them from TGDCs.

Cystic lymphangiomas (cystic hygromas) are typically multiloculated, infiltrative lesions that often cross fascial planes and involve multiple cervical spaces. They are most commonly diagnosed in pediatric patients and are relatively rare in adults. Imaging usually reveals a trans-spatial, septated cystic mass [13]. In contrast, the lesion in our patient was unilocular, well-defined, and confined to the infrahyoid midline region, without evidence of infiltration into adjacent cervical compartments, making lymphangioma an unlikely diagnosis.

Overall, the combination of a strictly midline infrahyoid location, homogeneous cystic morphology, absence of enhancement, and close association with the hyoid bone on computed tomography strongly supported the diagnosis of a giant TGDC in the present case, allowing reliable exclusion of other cystic cervical entities.

Typically, TGDCs measure less than 3 cm, with reported average diameters ranging from 2-4 cm [4]. Giant TGDCs are distinctly uncommon and are predominantly reported in adults, likely reflecting slow and indolent growth over many years. Our lesion measured 6.3 cm and weighed 117.5 g, placing it among the larger TGDCs described in the literature.

Giant TGDCs in adults may significantly alter midline cervical anatomy and mimic other pathological entities, thereby underscoring the importance of meticulous imaging-based evaluation prior to surgery in order to ensure accurate diagnosis and appropriate surgical strategy.

Table 1 provides a focused comparison of reported adult giant TGDCs, highlighting patient age, lesion size, imaging modality, and surgical management. Compared with previously published cases, the present report represents a rare instance of a giant TGDC in a middle-aged adult. This observation supports the concept that TGDCs may remain clinically silent for prolonged periods before progressively enlarging and becoming symptomatic in adulthood.

**Table 1:** Comparative summary of reported adult giant thyroglossal duct cysts.

Study	Age/sex	Location	Dimensions (cm)	Imaging	Surgical treatment
Baisakhiya [6]	65/M	Anterior, infrahyoid	11 × 9	CT/US	Sistrunk
El-Ayman et al. [1]	85/M	Anterior, infrahyoid	9.2 × 7.6	CT	Sistrunk
Mortaja et al. [4]	36/F	Anterior, infrahyoid	6 × 6	CT	Sistrunk
McNamara et al. [14]	85/F	Anterior + lingual	10 × 8	CT/MRI	Sistrunk + lingual excision
Marom et al. [15]	35/M	Anterior	30 × 24	CT	Sistrunk
Our case	37/F	Infrahyoid	6.3 × 4.8 × 3.3	CT	Sistrunk

Surgical excision remains the treatment of choice, with the Sistrunk procedure constituting the gold standard [8]. This technique involves en bloc removal of the cyst, the central portion of the hyoid bone, and the thyroglossal tract up to the foramen cecum, thereby minimizing recurrence. Reported recurrence rates following the Sistrunk procedure range from 3%-4%, markedly lower than those following simple cyst excision [16].

In a meta-analysis including 1371 patients, no major postoperative complications were observed, and infection was identified as the most common minor complication [8]. In our patient, surgery was uneventful, and no recurrence was noted during 1 year of follow-up, consistent with the excellent outcomes reported in the literature.

Several alternative surgical approaches have been described in the literature, particularly in selected or complex cases. Simple cyst excision without removal of the central portion of the hyoid bone has been historically performed. However, this approach is associated with significantly higher recurrence rates, reaching up to 55%, and is therefore no longer recommended [17]. Extended or modified Sistrunk procedures have been advocated in cases of large, recurrent, or suprahyoid–infrahyoid extending cysts, involving wider tract excision and meticulous dissection along the embryologic course of the thyroglossal duct.

Minimally invasive techniques, including endoscopic and robotic-assisted approaches, have also been reported, primarily for cosmetic purposes and in selected adult patients [18]. However, their role in the management of giant TGDCs remains limited and not well established.

Recent studies have demonstrated that anatomical variations may substantially influence surgical decision-making, particularly in head and neck pathologies, where distortion of normal landmarks can complicate both radiologic interpretation and operative planning [19].

In the present case, the large size of the lesion and its close relationship with the central portion of the hyoid bone were clearly demonstrated on preoperative imaging, allowing accurate diagnosis and appropriate surgical planning. Complete excision using the Sistrunk procedure resulted in an uneventful postoperative course and no recurrence at 1-year follow-up, supporting the effectiveness of this approach even in giant cysts.

This case emphasizes that giant thyroglossal duct cysts, although rare in adults, should be considered in the differential diagnosis of midline neck masses. Careful imaging-based evaluation is essential to avoid diagnostic pitfalls, and meticulous adherence to the Sistrunk procedure ensures excellent clinical outcomes, even in unusually large lesions.

## Conclusion

Giant thyroglossal duct cysts in adults are rare and may mimic other midline neck lesions due to distortion of normal anatomy. This case highlights the importance of preoperative imaging for accurate diagnosis and surgical planning, and reinforces that complete excision via the Sistrunk procedure remains safe and effective even for large lesions, helping to avoid misdiagnosis and recurrence.

## Ethical approval

Ethical approval was not required for this single-patient case report, as per our institutional policy. All procedures were conducted in accordance with the ethical standards of the institutional and national research committees, and with the principles of the Declaration of Helsinki.

## Patient consent

Written informed consent was obtained from the patient for publication of this case and any accompanying images.
